# Physical and functional measures predicting long-term mortality in community-dwelling older adults: a comparative evaluation in the Singapore Longitudinal Ageing Study

**DOI:** 10.18632/aging.203756

**Published:** 2021-12-11

**Authors:** Chin Yee Cheong, Philip Yap, Xinyi Gwee, Denise Q.L. Chua, Shiou Liang Wee, Keng Bee Yap, Tze Pin Ng

**Affiliations:** 1 Department of Geriatric Medicine, Khoo Teck Puat Hospital, Singapore; 2 Gerontology Research Programme, Department of Psychological Medicine, Yong Loo Lin School of Medicine, National University of Singapore, Singapore; 3 Health and Social Sciences Cluster, Singapore Institute of Technology, Singapore; 4 Department of Geriatric Medicine, Ng Teng Fong Hospital, Singapore

**Keywords:** functional measures, gait speed, gait measurement, mortality, community-dwelling

## Abstract

Measures of functional status are known to predict mortality more strongly than traditional disease risk markers in old adult populations. Few studies have compared the predictive accuracy of physical and functional measures for long-term mortality. In this prospective cohort study, community-dwelling older adults (*N* = 2906) aged 55 + (mean age 66.6 ± 7.7 years) were followed up for mortality outcome up to 9 years (mean 5.8 years). Baseline assessments included Timed Up-and-Go (TUG), gait velocity (GV), knee extension strength, Performance Oriented Mobility Assessment, forced expiratory volume in 1 second, Mini-Mental State Examination (MMSE), Geriatric Depression Scale, frailty, and medical morbidity. A total of 111 (3.8%) participants died during 16976.7 person-years of follow up. TUG was significantly associated with mortality risk (HR = 2.60, 95% CI = 2.05–3.29 per SD increase; HR = 5.05, 95% CI = 3.27–7.80, for TUG score ≥ 9 s). In multivariate analysis, TUG remained significantly associated with mortality (HR = 1.64, 95% CI = 1.20–2.19 per SD increase; HR = 2.66, 95% CI = 1.67–4.23 for TUG score ≥ 9 s). In multivariable analyses, GV, MMSE, Frailty Index (FI) and physical frailty, diabetes and multi-morbidity were also significantly associated with mortality. However, TUG (AUC = 0.737) demonstrated significantly higher discriminatory accuracy than GV (AUC = 0.666, *p* < 0.001), MMSE (AUC = 0.63, *p* < 0.001), FI (AUC = 0.62, *p* < 0.001), physical frailty (AUC = 0.610, *p* < 0.001), diabetes (AUC = 0.582, *p* < 0.001) and multi-morbidity (AUC = 0.589, *p* < 0.001).

TUG’s predictive accuracy shows surpassing predictive accuracy for long-term mortality in community-dwelling older adults.

## INTRODUCTION

Previous studies have shown that traditional clinical risk factors known to be associated with mortality risk, such as smoking, obesity, chronic diseases and comorbidity in the general adult population, lose their importance in old populations, typically older than 65 years. Instead, disability, poor physical and cognitive functions were more strongly predictive of mortality [1, 2]. This may be explained by the survival effect among individuals who remain alive while their contemporaries have already died in middle-age or younger-old age from smoking-, alcohol- and obesity-related diseases. Indeed, among very old people (over 75 s), paradoxical inverse mortality risks are sometimes found in association with obesity and cholesterol. Smokers, drinkers and obese individuals who survive into older age may perhaps have genetic and/or environmental characteristics that protect them against the toxic effects of harmful habits. Risk factors measured at old age may not reflect lifetime exposures since non-smoker and non-drinkers may have stopped their habits for health-related reasons, and there may have been significant weight changes previously.

Clinical measures of health and functional statuses such as cognition [3], depression [4], impaired pulmonary function [5], slow gait velocity [6] and frailty [7] have been investigated and consistently shown to predict mortality among older adults. These measures are not only related to specific chronic disease(s) or multi-morbidity, but also reflect the broad underlying intrinsic capacity of older people resulting from the interaction of physical and mental health declines. Few studies have evaluated various physical and functional measures together and compared their performance in predicting long-term mortality.

The Timed Up-and-Go test (TUG) is a widely used physical performance test of functional mobility in older persons, as it is easily performed without special equipment. It has high interrater and test-retest reliability [8]. TUG assesses static balance, dynamic balance, lower limbs strength [9, 10], and gait speed. Poor TUG performance has been linked to recurrent falls [11], impaired physical and cognitive function [12], poor quality of life [13], dementia [14] and frailty [15, 16]. Previous studies have shown that TUG predicts all-cause mortality of older adults [17–23].

In this study, we evaluated the predictive accuracy of TUG for long-term mortality and compared its performance with those of other commonly used measures of physical strength, balance and gait, functional mobility, global cognition and depression in a cohort of over-55-year-olds participating in the Singapore Longitudinal Aging Study 2 (SLAS-2) followed up for mortality risks up to 9 years (mean of 5.8 years). We hypothesized that the TUG has surpassing accuracy for predicting long-term mortality over gait velocity (measured on the fast gait test), knee extension strength (KES), the Performance-Oriented Mobility Assessment (POMA), the Mini-Mental State Examination (MMSE), depressive symptoms (measured by the Geriatric Depression Scale), forced expiratory volume - one second (FEV_1_), as well as frailty (Frailty Index and Physical Frailty phenotype) and multi-morbidity, which are two other clinical diagnoses known to predict mortality.

## RESULTS

The participants have a mean age of 66.6 ± 7.7 years. More than half of the participants were female (*n* = 1829, 62.9%), never smokers (*n* = 2276, 78.3%) and had hypertension (*n* = 1807, 62.2%). (Table 1) TUG was significantly correlated (*p* < 0.001) with GV (r = −0.593), KES (*r* = −0.238), POMA (r = −0.430) and MMSE (r = −0.326), as well as FEV1% (r = −0.133), GDS (r = 0.196), FI (r = 0.443), and physical frailty (r = 0.356). Up to 31 Dec 2016, a total of 111 (3.8%) participants died during a total follow up period of 16976.7 person-years. The principal causes of death were cancer (40%, including 12% lung cancer, 5% colorectal cancer), cardiovascular diseases (25%, including 10% stroke), pneumonia (15%), COPD (5%), kidney failure (2%).

We found that TUG was associated with significant mortality risk, whether it was analysed as a continuous variable or a binary categorical variable. The association of TUG and mortality in an unadjusted model has the HR of 2.6 (95% CI, 2.05–3.29, *p* < 0.001) per SD increase of TUG, and HR of 5.05 (95% CI, 3.27–7.80, *p* < 0.001) when analyzed as a binary variable (≥9 s vs. <9 s). It remained significantly associated with mortality even after adjusting for baseline sociodemographic, lifestyle risk factors, as well as comorbidities (HR = 1.64, 95% CI, 1.20–2.19, *p* < 0.001, per SD increase; HR = 2.66, 95% CI, 1.67–4.23, *p* < 0.001, TUG ≥9 s vs. <9 s). (Table 2A, 2B) When all physical and functional performance measure was analyzed simultaneously in the same model with all covariates, TUG remained significantly associated with mortality (HR = 1.45, 95% CI, 1.01–2.07, *p* < 0.05, per SD increase; HR = 2.02, 95% CI, 1.26–3.25, *p* = 0.004 with TUG binary score). In contrast, other physical and functional performance measures were no longer significantly associated with mortality per SD score increase, except for low KES (HR = 1.80, 95% CI, 1.20–2.69, *p* = 0.004) (Table 3).

**Table 1:** Characteristics of study participants in the Singapore Longitudinal Ageing Study (SLAS-2) cohort (*N* = 2906).

**Characteristics**		**Mean or %**	**± SD or (*N*)**	**Skewness**
Age	Mean ± SD	66.6	± 7.7	
Sex	Female	62.9	(1829)	
Ethnicity	Chinese	87.7	(2549)	
	Non-Chinese (Malay, Indian and Others)	12.3	(357)	
Education	None	19.4	(563)	
	1–6 years	43.2	(1254)	
	>6 years	37.5	(1089)	
Housing type	Low-end 1–2 rooms	21.3	(619)	
	3 rooms	28.5	(827)	
	≥4 rooms and others	50.2	(1460)	
Live alone		14.6	(424)	
Smoking	Never	78.3	(2276)	
	Ex-smoker	11.8	(344)	
	Current smoker	9.8	(286)	
BMI, kg/m^2^	Mean ± SD	24.2	± 4.1	
<18.5	Underweight	5.5	(159)	
18.5–29.9	Non-obese	84.4	(2453)	
≥30	Obese	7.5	(219)	
Waist circumference, cm (men)	Mean ± SD	88.6	± 9.9	
(women)	Mean ± SD	83.2	± 10.5	
Central obesity (men)	Yes vs. no	43.5	(469)	
(women)	Yes vs. no	61.6	(1127)	
Physical activity score	1–12	6.2	± 1.5	
Social activity score	6–24	11.1	± 2.6	
Productive activity score	4–16	9.9	± 1.9	
Multi-morbidity	≥5 vs. 0–4	18.1	(525)	
Heart disease	Yes vs. no	8.8	(257)	
Stroke	Yes vs. no	3.5	(102)	
Diabetes	Yes vs. no	20.2	(587)	
Hypertension	Yes vs. no	62.2	(1807)	
Chronic kidney disease	Yes vs. no	8.4	(244)	
TUG	Mean ± SD	8.9	± 3.7	4.57
GV (Reversed)	Mean ± SD	1.3	± 0.35	0.01
KES (Reversed)	Mean ± SD	16.4	± 6.7	1.08
POMA (Reversed)	Mean ± SD	25.6	± 1.6	−6.90
FEV1% (Reversed)	Mean ± SD	104.4	± 23.0	−0.18
MMSE (Reversed)	Mean ± SD	27.8	± 2.8	−2.44
GDS	Mean ± SD	0.74	± 1.48	4.38
Frailty Index	Mean ± SD	0.10	± 0.06	1.65
Physical frailty	Prefrail vs. robust	43.8	(1274)	
	Frail vs. robust	4.9	(141)	
TUG	≥9	31.8	(923)	
POMA	<25	8.4	(224)	
GV	>1.0 m/s	18.5	(539)	
KES	<15 kg (M), <11 kg (F)	30.0	(871)	
FEV_1_%	<70%	8.5	(248)	
MMSE	≤23	7.6	(2.21)	
GDS	≥5	2.5	(73)	
Frailty Index	≥0.15	17.1	(498)	
Physical frailty	1–5	48.7	(1415)	

Abbreviations: TUG: Timed Up-and-Go; POMA: Performance Oriented Mobility Assessment; GV: gait velocity; KES: knee extension strength; FEV1%: forced expiratory volume in 1 second in percentage; MMSE: Mini-Mental State Examination; GDS: Geriatric Depression Scale.

**Table 2A:** Hazard ratio estimates of physical and functional performance and chronic disease markers predicting mortality.

**Predictor variable**	**Measurement Units**	**Unadjusted**			**Model 1**			**Model 2**		
		**HR**	**95% CI**	***P***	**HR**	**95% CI**	***P***	**HR**	**95% CI**	***P***
Per SD or equivalent										
TUG	Per SD increase	2.60	(2.05, 3.29)	^***^	1.85	(1.42, 2.42)	^***^	1.64	(1.20, 2.19)	^***^
GV (Reversed)	Per SD increase	1.83	(1.48, 2.27)	^***^	1.47	(1.18, 1.85)	^***^	1.33	(1.04, 1.69)	^*^
KES (Reversed)	Per SD increase	1.24	(1.01, 1.53)	^*^	1.46	(1.15, 1.86)	^**^	1.38	(1.07, 1.78)	^*^
FEV1% (Reversed)	Per SD increase	1.24	(1.02, 1.51)	^*^	1.29	(1.06, 1.56)	^**^	1.15	(0.94, 1.41)	
POMA (Reversed)	Per SD increase	1.47	(1.09, 1.97)	^**^	1.18	(0.87, 1.59)		1.04	(0.75, 1.44)	
MMSE (Reversed)	Per SD increase	1.65	(1.31, 2.08)	^**^	1.32	(1.02, 1.70)	^*^	1.20	(0.91, 1.60)	
GDS	Per SD increase	1.42	(1.08, 1.87)	^**^	1.28	(0.97, 1.68)		0.96	(0.71, 1.28)	
Frailty Index	Per SD increase	1.70	(1.38, 2.08)	^***^	1.41	(1.13, 1.76)	^**^	1.18	(0.90, 1.53)	
Physical frailty	Per point score	1,53	(1.29, 1.81)	^***^	1.27	(1.07, 1.11)	^***^	1.12	(0.92, 1.37)	
Age	Per SD increase	2.46	(1.97, 3.06)	^***^	2.34	(1.88, 2.92)	^***^	1.75	(1.34, 2.28)	^***^
Per binary variables										
TUG	> = 9 vs. <9 s	5.05	(3.27, 7.80)	^***^	3.28	(2.06, 5.22)	^***^	2.66	(1.67, 4.23)	^***^
GV	<1.0 m/s	2.81	(1.91, 4.12)	^***^	1.83	(1.21, 2.77)	^**^	1.69	(1.08, 2.63)	^*^
KES	15 kg (M), 11 kg (F)	2.58	(1.78, 3.75)	^***^	2.06	(1.41, 3.01)	^***^	2.02	(1.36, 3.01)	^***^
FEV_1_%	<70% vs. ≥70%	2.10	(1.29, 3.39)	^**^	2.15	(1.32, 3.53)	^**^	1.84	(1.09, 3.10)	^*^
POMA	24/25	2.20	(1.35, 3.57)	^**^	1.57	(0.96, 2.58)		1.39	(0.82, 2.38)	
MMSE	≤23 vs. ≥24	2.51	(1.54, 4.08)	^***^	1.53	(0.90, 2.57)		1.28	(0.73, 2.23)	
GDS	≥5 vs. <5	1.68	(1.13, 2.38)	^**^	1.66	(0.68, 4.09)		0.76	(0.27, 2.13)	
Frailty Index	≥0.15 vs. <0.15	2.99	(2.03, 4.39)	^***^	2.13	(1.41, 3.22)	^***^	1.75	(1.08, 2.82)	^*^
Physical frailty	0 vs. 1–5	2.56	(1.41, 4.67)	^**^	1.89	(1.26, 2.82)	^**^	1.58	(1.01, 2.47)	^*^
Age	≥75 vs. <75	4.66	(3.21, 6.78)	^***^	4.24	(2.91, 6.17)	^***^	2.74	(1.75, 4.31)	^***^

Model 1: adjusted for age (per year) and sex. Model 2: adjusted for covariates in Model 1 (age and sex) + education, housing status, living alone, physical activity, social activity, productive activity, smoking, BMI, central obesity, heart disease, stroke, diabetes/prediabetes, hypertension, chronic kidney disease and multimorbidity.

**Table 2B:** Hazard ratio estimates of chronic disease and behavioural risk markers predicting mortality with base model co-variables.

**Other clinical predictors and base model variables**		**Unadjusted**			**Model 1**			**Model 2**		
		**HR**	**95%CI**	***P***	**HR**	**95% CI**	***P***	**HR**	**95% CI**	***P***
Smoking	Past (vs. Never)	4.07	(2.63, 6.28)	^***^	2.23	(1.34, 3.69)	^**^	2.01	(1.20, 3.39)	^**^
	Current (vs. Never)	3.59	(2.23, 5.78)	^***^	2.88	(1.71, 4.86)	^***^	2.53	(1.46, 4.37)	^**^
BMI, kg/m^2^	18.5–29.9 (vs. <18.5)	0.61	(0.32, 1.18)		0.74	(0.38, 1.42)		0.82	(0.41, 1.65)	
	≥30 (vs. <18.5)	0.30	(0.09, 0.97)	^*^	0.49	(0.15, 1.56)		0.52	(0.15, 1.81)	
Central obesity	Yes vs. no	0.64	(0.44, 0.93)	^*^	0.74	(0.50, 1.08)		0.77	(0.50, 1.17)	
Hypertension	Yes vs. no	2.11	(1.35, 3.29)	^**^	1.28	(0.81, 2.03)		1.27	(0.75, 2.15)	
Diabetes	Yes vs. no	2.16	(1.46, 3.17)	^***^	1.87	(1.27, 2.76)	^**^	1.81	(1.11, 2.94)	^*^
Heart disease	Yes vs. no	2.40	(1.49, 3.86)	^***^	1.66	(1.03, 2.68)	^*^	1.48	(0.86, 2.53)	
Stroke	Yes vs. no	2.05	(0.99, 4.20)		1.41	(0.68, 2.90)		1.09	(0.50, 2.36)	
Chronic kidney disease	Yes vs. no	2.60	(1.63, 4.15)	^***^	1.18	(0.72, 1.94)		0.82	(0.48, 1.40)	
Multi-morbidity	≥5 vs. 0–4	2.48	(1.68, 3.66)	^***^	1.69	(1.12, 2.52)	^**^	1.08	(0.62, 1.88)	
Age	Single year	1.10	(1.07, 1.21)	^***^	1.09	(1.07, 1.12)	^***^	1.08	(1.05, 1.10)	^***^
Male sex		2.70	(1.84, 3.95)	^***^	2.39	(1.63, 3.51)	^***^	0.78	(0.46, 1.31)	
Education	1–6 years vs. >6 years	1.32	(0.83, 2.07)		1.07	(0.67, 1.70)		0.96	(0.59, 1.56)	
	None vs. >6 years	1.86	(1.13, 3.06)	^*^	1.22	(0.70, 2.12)		0.94	(0.52, 1.70)	
Housing type	3 room vs. 4 + room	1.60	(0.96, 2.66)		1.28	(0.77, 2.14)		0.98	(0.57, 1.67)	
	1–2 room vs. 4 + room	3.49	(2.23, 5.44)	^***^	2.14	(1.35, 3.40)	^***^	1.64	(0.97, 2.76)	
Live alone	Yes vs. no	1.43	(0.90, 2.26)		1.24	(0.78, 1.97)		0.93	(0.55, 1.57)	
Physical activity score	Per point score	0.80	(0.70, 0.92)	^**^	0.83	(0.72, 0.96)	^**^	0.92	(0.79, 1.09)	
Social activity score	Per point score	0.87	(0.80, 0.95)	^***^	0.90	(0.83, 0.98)	^*^	0.94	(0.86, 1.03)	
Productive activity score	Per point score	0.72	(0.65, 0.80)	^***^	0.84	(0.75, 0.93)	^**^	0.92	(0.82, 1.03)	

Abbreviation: HR: hazard ratio; ^*^p < 0.05; ^**^p < 0.01; ^***^P < 0.001. Model 1: adjusted for age (per year) and sex. Model 2: adjusted for covariates in Model 1 (age and sex) + education, housing status, living alone, physical activity, social activity, productive activity, smoking, BMI, central obesity, heart disease, stroke, diabetes/prediabetes, hypertension, chronic kidney disease and multi-morbidity. Hazard ratios are unadjusted for functional predictors; Binary cut-offs shown are commonly used in previous research and clinical applications.

**Table 3:** Hazard ratios of association with mortality for physical and functional performance measures simultaneously present in the same model.

**Measure**		**Model 1**			**Model 2**		
		**HR**	**95% CI**	***P***	**HR**	**95% CI**	***P***
Standard deviation score							
TUG	Per SD increase	2.17	(1.55, 3.04)	^***^	1.45	(1.01, 2.07)	^*^
GV (Reversed)	Per SD increase	1.10	(0.82, 1.46)		1.02	(0.75, 1.38)	
KES	Per SD increase	0.90	(0.72, 1.23)		1.27	(0.98, 1.66)	
POMA	Per SD increase	0.84	(0.61, 1.16)		0.86	(0.61, 1.21)	
MMSE (Reversed)	Per SD increase	1.15	(0.89, 1.49)		1.07	(0.80, 1.43)	
GDS	Per SD increase	1.04	(0.78, 1.39)		0.94	(0.70, 1.27)	
Frailty Index	Per SD increase	1.18	(0.90, 1.54)		1.08	(0.81, 1.44)	
Physical frailty	Per point increase	1.12	(0.91, 1.54)		0.98	(0.80, 1.21)	
Binary score							
TUG	> = 9 vs. <9 s	2.74	(1.75, 4.30)	^***^	2.02	(1.26, 3.25)	^**^
GV	<1.0 m/s	0.78	(0.49, 1.25)		1.16	(0.72, 1.88)	
KES	15 kg (M), 11 kg (F)	1.78	(1.20, 2.65)	^**^	1.80	(1.20, 2.69)	^**^
POMA	24/25	1.15	(0.68, 1.94)		1.02	(0.59, 1.76)	
MMSE	≤23 vs. ≥24	0.80	(0.47, 1.35)		1.02	(0.58, 1.79)	
GDS	≥5 vs. <5	0.93	(0.37, 2.34)		0.84	(0.32, 2.16)	
Frailty Index	≥0.15 vs. <0.15	1.71	(1.08, 2.70)	^*^	1.50	(0.25, 1.10)	
Physical frailty	3–5 vs. 0–2	0.73	(0.37, 1.44)		0.50	(0.25, 1.01)	

Abbreviation: HR: hazard ratio; ^*^p < 0.05; ^**^p < 0.01; ^***^p < 0.001. Model 1: All physical and functional performance measure included in the same model together. Model 2: All physical and functional performance measure included in the same model together with covariates (age, sex, education, housing status, living alone, smoking, physical activity, social activity, productive activity, heart disease, stroke, diabetes/prediabetes, hypertension, chronic kidney disease and multi-morbidity). Binary cut-offs shown are commonly used in previous research and clinical applications.

**Figure 1:**
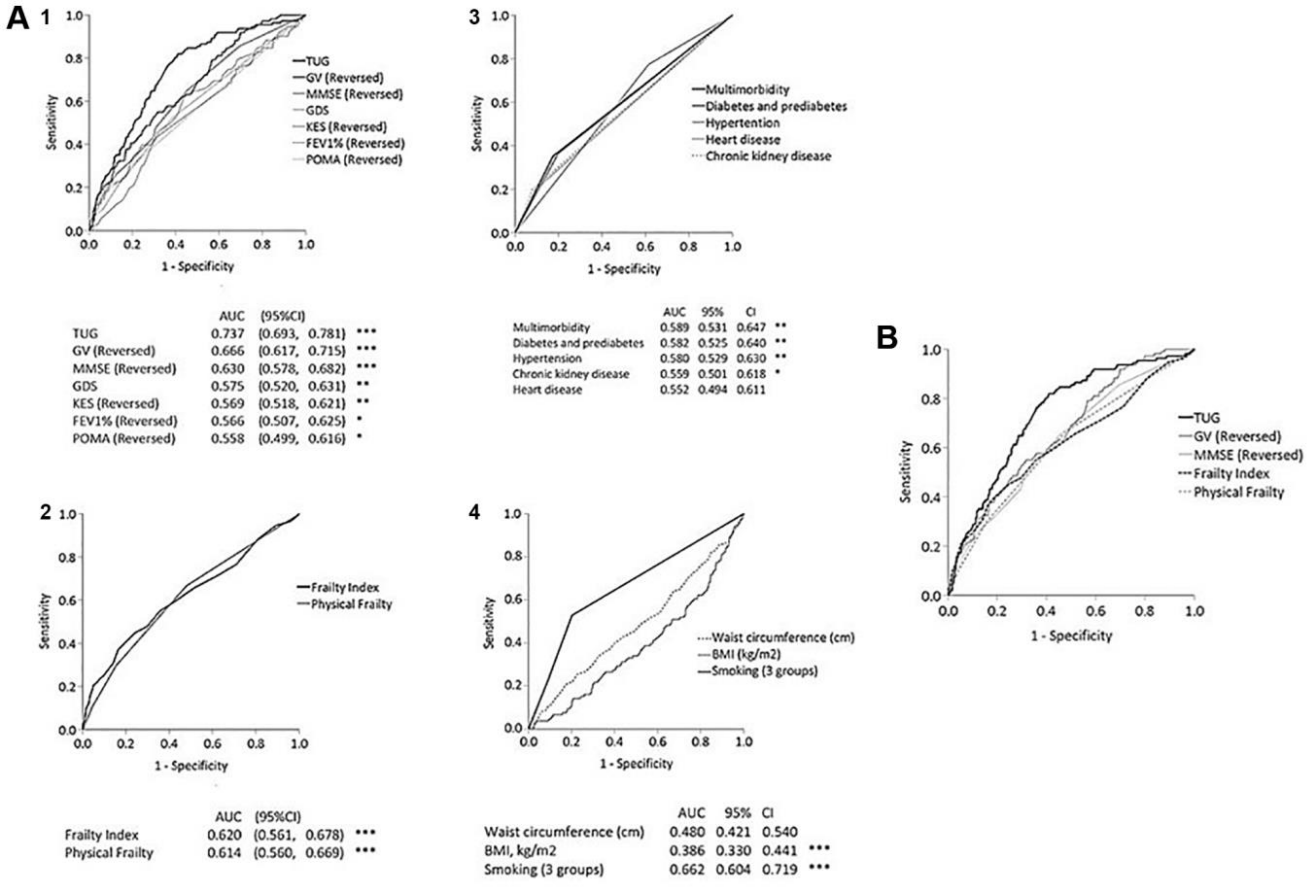
(**A**) Receiver operating curves of 1-year mortality prediction by physical and functional tests (panel 1), frailty index and physical frailty (panel 2), chronic disease and multi-morbidity (panel 3), and smoking, BMI and waist circumference (panel 4). (**B**) Receiver operating curves of 10-year mortality prediction by TUG, GV, MMSE, FI and physical frailty.

TUG (AUC = 0.737) demonstrated a significantly higher predictive accuracy for mortality than GV (AUC = 0.666, *p* < 0.001), MMSE (AUC = 0.63, *p* < 0.001), FI (AUC = 0.620, *p* < 0.001) and physical frailty (AUC = 0.610, *p* < 0.001). (Table 4 and Figure 1) AUCs for chronic diseases and multi-morbidity were between 0.552 and 0.589, smoking was 0.662, BMI and central obesity were 0.386 and 0.480. The AUC for age was 0.730 (95% CI, 0.681–0.778).

**Table 4:** Predictive accuracy of TUG for mortality compared to gait velocity, frailty index, and physical frailty.

**Measures**	**AUC**	**95% CI**		***p***	**Cut-off**	**Sensitivity**	**Specificity**	**PPV**	**NPV**
TUG	0.737	(0.693, 0.781)	(a)	^***^	8.0 s	0.856	0.488	0.062	0.988
			(b)	^***^	9.0 s	0.656	0.696	0.079	0.981
			(c)	^***^	10.0 s	0.468	0.804	0.087	0.974
			(d)	^***^	11.0 s	0.351	0.870	0.097	0.971
					12.0 s	0.261	0.918	0.102	0.969
GV (Reversed)	0.666	(0.617, 0.715)	(b)	^***^	0.8 m/s	0.189	0.933	0.102	0.967
					0.9 m/s	0.261	0.891	0.087	0.968
					1.0 m/s	0.378	0.822	0.078	0.971
					1.1 m/s	0.477	0.740	0.068	0.973
					1.2 m/s	0.559	0.651	0.060	0.974
					1.3 m/s	0.631	0.547	0.052	0.974
					1.4 m/s	0.802	0.415	0.052	0.981
MMSE (Reversed)	0.630	(0.578, 0.682)	(c)	^***^	18/19	0.045	0.983	0.096	0.963
					23/24	0.180	0.928	0.090	0.966
					26/27	0.324	0.816	0.062	0.968
					28/29	0.649	0.549	0.054	0.975
Frailty Index	0.620	(0.561, 0.678)	(a)	^***^	0.070	0.766	0.390	0.041	0.969
					0.080	0.712	0.480	0.044	0.971
					0.090	0.658	0.482	0.048	0.973
					0.150	0.369	0.836	0.082	0.971
					0.210	0.207	0.949	0.139	0.968
					0.250	0.117	0.974	0.151	0.965
Physical frailty	0.610	(0.560, 0.669)	(d)	^***^	0/1–5	0.667	0.520	0.052	0.975
					0–2/3–5	0.108	0.954	0.085	0.964

^***^p < 0.001, (a) TUG vs. Frailty index; (b) TUG vs. GV; (c) TUG vs. MMSE; (d) TUG vs. Physical frailty. Binary cut-offs shown cover a range commonly used in previous research and clinical applications. Only predictors with AUC above 0.60 were shown. Abbreviations: AUC: area under the curve; PPV: positive predictive value; NPV: negative predictive value. AUC for age: 0.730 (95% CI: 0.681–0.778)

A TUG cut-off of 8.0 s was associated with high sensitivity to 0.856 (with low specificity of 0.488), and TUG of 10.0 s was associated with high specificity of 0.804 (with low sensitivity of 0.468). A TUG cut-off of 9.0 s was associated with optimal sensitivity and specificity of 0.656 and 0.696, respectively.

We conducted further stratified analyses by sex and age groups and found consistent associations and similar predictive accuracy for both men and women and younger (<75) and older (≥75) individuals (Supplementary Tables 1 and 2).

## DISCUSSION

In this study, we re-capitulated previous observations that physical and functional measures predict mortality risk. Notably, we showed that TUG, gait speed, KES, FEV_1_, and frailty were significantly associated with increased mortality, even after adjusting for sociodemographic, lifestyle, and traditional disease and health behavioural risk markers. Diabetes, cardiovascular disease, and multi-morbidity were also associated with increased mortality risks but low predictive accuracy in this cohort. Notably, compared with standardized units using their SD value, TUG showed the strongest hazard ratio for mortality risk among physical and functional measures. The AUC’s for all measures clearly showed that the discriminant accuracy for predicting mortality risk was highest for TUG. The finding remained consistent, whether the TUG was analyzed as a continuous variable or a binary categorical variable with the cut-off of 9 s.

Previous studies have reported similarly that TUG predicts mortality [17–23]. Among them, three studied only men [17] or women [18, 19]; one studied middle-aged postmenopausal women [19]; three were Asian studies [21–23], of which one evaluated short-term 2-year mortality risk [22], and one evaluated cardiovascular mortality [23]. Only a few studies, beside our study, evaluated TUG alongside other physical or functional measures: one study evaluated two measures (TUG and handgrip strength) [22], another study evaluated four measures (TUG, handgrip strength, five times sit-to-stand test, standing balance) [17], and another study also evaluated four measures (TUG, usual gait velocity, functional reach, one-leg stance) [18]. Our finding showing TUG to have surpassing predictive accuracy for long-term mortality is consistent with the findings reported of older men aged 71–86 in Belgium [17], and another cohort of older men and women aged 65–94 in Singapore [22]. However, differing results were reported by Idland et al., who followed up a small group of 300 community-dwelling older women (mean age 80.9 years) for 13.5 years showing that usual gait velocity was the strongest predictor for all-cause mortality [18]. We performed stratified analyses by sex and found consistent associations and predictive accuracy for both men and women.

The TUG is a complex test of functional mobility that reflects strength, balance and mobility through assessing the ability to transfer, sit-to-stand, walk, and turn [9, 10]. The sit-to-stand component includes a sequence of multiple subtasks, requiring forward movement of the centre-of-mass while still seated (preparatory to standing), acceleration of the centre-of- mass in the anterior-posterior and vertical plane, push-off, and stabilization once standing is achieved. The walking component requires appropriate initiation of stepping, acceleration and deceleration, and preparation to turn twice. The first turning sequence and the final turning around to sit down requires some level of planning, orientation in space and organization. The transfer and turning components are thus cognitively demanding, particularly on tasks of executive function [24].

The significant correlations between TUG and other physical and functional measures suggest that they have overlapping and non-overlapping domains of physical, cognitive and functional performance with each other. TUG is less correlated with muscle strength (KES) than with gait speed. This is in accord with observations [25] that muscle strength partially determines variations in gait performance, besides other determinants such as reaction time, balance, and proprioception. Furthermore, physical performance tests decline faster than muscle decline in the older population [26]. GV was also shown in this study to be more strongly predictive of mortality than muscle strength.

Muscle strength and gait speed are recommended for diagnosing sarcopenia and assessing its severity, respectively [27]. TUG’s strong association with mortality is likely due to its ability to identify sarcopenia and frailty; both documented to predict mortality [7, 28]. Sarcopenia, involving the accelerated loss of generalized skeletal muscle mass and function, is considered a precursor and component [29, 30] of frailty, which increases the vulnerability to adverse health outcomes. Sarcopenia is about twice as common as frailty, depending on the criteria used [29]; hence not all sarcopenic older people are frail. Two widely accepted operational conceptualizations of frailty are used in this study: the FI considers the cumulative deficits from all diagnosable health conditions; the other physical phenotype of frailty is more closely related to sarcopenia but includes inactivity and exhaustion as additional criteria. Per other studies [7], FI appears to be a stronger predictor of mortality in this study.

Taken together, TUG thus provides more information in a single test than GV, POMA, FEV1 or MMSE alone. It also shows a surpassing accuracy than these physical and functional tests, as well as known disease and health risk markers in predicting mortality. Among the latter, only smoking showed a relatively high AUC of 0.662, whereas BMI and central obesity showed AUCs significantly below 0.50, consonant with their well-known paradoxical ‘protective’ effect on mortality that has been reported in numerous studies [31]. On the other hand, age showed a higher AUC of 0.730. Although the TUG appears to have only marginally higher AUC than age in predicting mortality, this does not detract from its potential clinical utility. TUG differs from age in being a modifiable risk predictor that provide clinically useful information for targeted intervention to reduce mortality risk.

### TUG cut-off

Our results align with previous studies showing a monotonic increase of mortality risk per SD increase in TUG [28]. There is no recommended cut-off for mortality prediction. Various optimal cut-off points are recommended specifically for different predicted adverse outcomes and different population groups of healthy and unwell persons. For example, the American Geriatrics Society (AGS) and British Geriatrics Society (BGS) guidelines recommended a TUG cut-off of 13.5 s for fall risk prediction of community-dwelling older adults [32]. Asian older adults have a lower TUG than the Caucasian population due to the differences in habitual gait speed [33]. Two studies of Japanese and Singaporean older adults suggest appropriate cut-offs of 9.0 s or 9.5 s for ADL disability risk among Asians [33, 34]. Consistent with these studies, TUG cut-off of 9.0 s gave the optimal balance of sensitivity (0.656) and specificity (0.696), whereas a cut-off of 8.0 s increases the sensitivity to 0.856 (while lowering the specificity to 0.488), and a higher cut-off of 10.s increases the specificity to 0.804 (while lowering the sensitivity to 0.468).

### Clinical implications

Our findings contribute to a greater appreciation of the TUG as a powerful clinical tool predicting not only physical and cognitive impairment, sarcopenia, frailty, and other adverse health outcomes [11–15], but long-term mortality as well. The TUG appears unique among other physical and functional measures commonly explored for use as prognostication tools in clinical research and practice. Its overall discriminant accuracy for mortality (AUC = 0.737) is no less than other accepted risk prediction or prognostication tools such as the Framingham risk index for cardiovascular disease mortality (AUC = 0.61) [35] or the BODE score for chronic obstructive pulmonary disease (AUC = 0.71) [36].

Further studies should explore whether combinations of clinical and functional markers could improve its prognostication value. Already, the TUG has been recommended by the AGS and BGS guidelines for fall risk prediction of community-dwelling older adults. As such there is broader justification for routine screening with the TUG (cut-off of ≥9 s) for early comprehensive assessment and intervention, particularly with clinical consideration of patients’ life expectancy during shared clinical decision making regarding chronic disease management, major surgeries and cancer screening.

### Strengths and limitations

Our study is uniquely able to evaluate the TUG alongside many clinical measures of physical and functional health status to compare their relative strengths and limitations for clinical use. We could do this in a large sample community-based cohort with diverse demographic, socio-economic and health characteristics. Follow up over 10 years for mortality was complete using computerized search for deaths via the National Death Registry. The results are reasonably generalizable to other Asian populations, but additional studies in other non-Asian ethnic populations should be conducted.

## MATERIALS AND METHODS

### Participants and setting

The Singapore Longitudinal Ageing Study is a prospective population-based study of ageing and health transitions of older adults aged 55 and above in Singapore. The current SLAS-2 study cohort was recruited between 2009 and 2013 from the South West and South Central regions of Singapore. A total of 3270 recruited participants underwent assessments for an extensive range of psychosocial, lifestyle and behaviour, medical, biological, physiological, diet and nutrition, physical and neurocognitive functioning, and health status variables. Previous publications have described the details of the participants’ recruitment and measurements [37]. The present study involved 2906 participants who provided baseline data who were followed up to 9 years (mean of 5.8) years for mortality. Participants who were not included in the mortality follow-up study did not have complete baseline data for physical, cognitive, and functional tests and did not differ substantially in baseline characteristics from the participants in this study. The study was approved by the National University of Singapore Institutional Review Board, and written informed consent was obtained.

### Baseline measurements

#### Physical and functional performance

*Timed Up-and-Go (TUG)* was measured by the time taken by the participant to stand up from an armchair (46 cm height), walk 3 metres, turn, walk back to the chair, and sit down again. The participants wore their regular footwear and used their customary walking aid, if required. Participants walked at their fastest pace with no physical assistance given. The test was administered twice, and the best performance time was used [8]. Various TUG cut-offs have been proposed or recommended for falls or disability prediction specific for different populations, and there are no suggested TUG cut-offs for mortality prediction. Asians generally have shorter mean TUG (faster gait speed) than Caucasians [33, 34]. We used an optimal TUG cut-off of 9.0s from receiver operating characteristics (ROC) analyses, consistent with a recommended cut-off of 9.0 s predicting disability in Japanese older adults [38].

*Gait velocity (GV)* was measured by the time in seconds taken for the participant to walk 6 metres at their fastest pace, averaged for two trials. Participants performed the test with a dynamic start on a smooth, flat 10-metre walkway with red-tape markers placed at the 0-, 2-, 8, and 10-metre points along the walkway, allowing for acceleration the first 2 metres and deceleration over the last 2 metres. The timing made a stopwatch is started when the toes of the leading foot cross the 2-meter mark and stopped when the toes of the leading foot cross the 8-meter mark. Cut-offs for Asians of <1.0 m/s has been recommended by previous studies [39].

*Knee extension strength (KES)* was measured for the lower limb maximum isometric strength. It was measured with the participant seated, the hip and knee angles at 90° using the strap and strain gauge component of the Physiological Profile Assessment [40], using three trials’ dominant leg average value (in kilograms). Cut-offs of 15 kg for males and 11 kg for females based on the lowest quintile value stratified by sex, were used to define low KES [41].

The *Performance Oriented Mobility Assessment (POMA)* battery measures both static and dynamic balance, with a separate subtest for balance and gait [42]. POMA is commonly used to predict falls and mortality of older adults [43, 44]. A cut-off score of <25 indicates a medium to high fall risk.

The *Geriatric Depression Scale 15-items (GDS-15)* score (0–15) was used to identify the presence of depressive symptoms (GDS ≥5) [45], and the *Mini-Mental State Examination (MMSE)* was used to assess global cognition and identify cognitive impairment (MMSE <23) [46]. Pulmonary function was assessed with the forced expiratory volume in 1 second (FEV1). FEV_1_ below 70% of the value predicted by age, sex, ethnicity, and height using local population equations indicates airflow obstruction.

#### Frailty

Two widely accepted models were used to measure the frailty status of the participants:

- Frailty Index (FI) [47]: a cumulative deficit model based on counts of dysfunction and impairment across multiple body systems. A total of 98 non-laboratory based evaluable health deficits were used to construct the index, expressed as a fractional value (number of observed deficits/number of evaluable deficits) from 0 (extremely robust) to 1 (extremely frail) (Supplementary Table 3). FI was analyzed as a continuous variable and binary variable using a cut-off of 0.15 and more to define frailty, based on calculations of stratum-specific likelihood ratios to determine the most appropriate cutoff to discriminate between frailty and non-frailty in predicting mortality in this cohort [44].
- Physical frailty: a physical phenotype model used in the Cardiovascular Health Study [48]. We used 5 operationally modified measures described in our previous study [41] for assessing shrinking, weakness, slowness, exhaustion and low activity. One point was assigned for the presence of each of the components, and the total summed score (from 0 to 5) was used to categorize participants as robust (0 points), prefrail (1–2 points) and frail (3–5 points).

#### Covariates

We collected baseline information such as age, sex and years of education. Participants’ housing type: low-end 1–2 room public housing apartments, 3 rooms or a higher-end with 4 rooms or others was used as an indicator of socio-economic status based on the Singapore population census data [49]. Lifestyle factors included participation in 16 categories of physical, social and productive activities described in a previous publication to derive aggregate score based on the number of activities and frequency of participation (on a 5-point Likert scale), with a higher score representing a higher level of participation [50].

### Mortality assessment

Participants’ mortality status from baseline up to 31 Dec 2016 was determined using the participants’ unique National Registration Identity Card number for computerized record linkage with the National Death Registry through the National Disease Registry Office of the Ministry of Health.

### Statistical analysis

We used Cox proportional hazard models to evaluate the association of TUG, other physical and functional measures, and chronic disease and behavioural risk markers (multi-morbidity, heart disease, diabetes mellitus, hypertension, chronic kidney disease, smoking, BMI, central obesity, frailty index, physical frailty) with mortality in a crude model and two adjusted models. In Model 1, the mortality HR estimate associated with each predictor variable was adjusted for age and sex (but not for ethnicity, as no deaths were observed among the small numbers of non-Chinese participants). Model 2 further adjusted for covariates in Model 1 as well as for education, housing status, living alone, smoking (but not alcohol, due to small sample size), physical activity, social activity, productive activity, heart disease, stroke, diabetes, hypertension, chronic kidney disease and multi-morbidity. Hazard ratios (HR) and 95% confidence intervals (95%CI) were estimated for each physical, functional and clinical predictor as a continuous variable and binary variable. The mortality HR value is variable for different cut-offs along with the range of values of the same predictor variable and for different measurement units of different predictor variables. Thus, for a valid comparison of the strengths of association with mortality between different predictors, we used a standardized approach to show per standard deviation (SD) increment of mortality HR.

The measures in predicting mortality were evaluated using receiver operating characteristic (ROC) curves, and the areas under the curves (AUCs) were compared using the DeLong’s method for significance testing [49]. An AUC between 0.7 and 0.8 is considered acceptable discrimination, between 0.8 and 0.9 is deemed excellent discrimination, and more than 0.9 is outstanding discrimination [51]. The discriminant accuracy of various optimal cut-off values was expressed as sensitivity, specificity, positive predictive value, and negative predictive values. Analysis of the data was performed using IBM SPSS version 25.

## CONCLUSIONS

Our study highlights the superior accuracy of TUG compared to other physical and functional measures in predicting long-term mortality among community-dwelling older adults. Taken together with evidence of the ability of the TUG to predict falls and other adverse health outcomes, the TUG appears to be uniquely positioned for use in early comprehensive geriatric assessment, and particularly in regard to shared clinical decision making requiring the prognostication of future life expectancy.

## Supplementary Materials

Supplementary Tables
