# Supplementary material for: Physical and functional measures predicting long-term mortality in community-dwelling older adults: a comparative evaluation in the Singapore Longitudinal Ageing Study
Source: Aging (Albany NY). 2021 Dec 11;13(23):25038–54. doi: 10.18632/aging.203756 (PMC8714162; doi:10.18632/aging.203756)
Supplement: Supplementary Tables [file aging-13-203756-s001.pdf]

SUPPLEMENTARY TABLES

Supplementary Table 1. Sex- and age-stratified analyses of hazard ratios of association with mortality for physical and functional performance measures simultaneously present in the same model.

Measure		Men			Women			Age < 75			Age ≥ 75		
		**HR**	**95% CI**	***P***	**HR**	**95% CI**	***P***	**HR**	**95% CI**	***P***	**HR**	**95% CI**	***P***
Standard deviation score													
TUG	Per SD increase	1.77	(1.15, 2.73)	**	2.35	(1.31, 4.22)	**	1.96	(1.26, 3.06)	**	1.85	(1.07, 3.19)	*
GV (Reversed)	Per SD increase	0.76	(0.54, 1.09)		1.26	(0.76, 2.08)		0.78	(0.55, 1.13)		1.34	(0.83, 2.17)	
KES	Per SD increase	1.39	(1.02, 1.91)	*	1.02	(0.67, 1.55)		0.80	(0.60, 1.06)		1.18	(0.80, 1.73)	
POMA	Per SD increase	0.76	(0.49, 1.17)		0.96	(0.60, 1.55)		0.82	(0.50, 1.36)		0.89	(0.57, 1.38)	
MMSE (Reversed)	Per SD increase	1.12	(0.78, 1.60)		1.51	(1.00, 2.26)	*	0.87	(0.58, 1.31)		1.20	(0.84, 1.71)	
GDS	Per SD increase	0.87	(0.60, 1.28)		1.23	(0.78, 1.92)		1.03	(0.68, 1.55)		1.13	(0.74, 1.71)	
Frailty Index	Per SD increase	1.20	(0.85, 1.69)		1.20	(0.78, 1.86)		1.27	(0.88, 1.82)		0.94	(0.64, 1.39)	
Physical frailty	Per point increase	0.91	(0.69, 1.19)		1.34	(0.96, 1.85)		1.07	(0.78, 1.47)		1.12	(0.86, 1.46)	
Binary score													
TUG	>=9 vs. <9 s	3.35	(1.90, 5.90)	***	1.59	(0.74, 3.44)		2.77	(1.55, 4.95)	***	1.41	(0.68, 2.92)	
GV	<1.0 m/s	0.83	(0.45, 1.52)		0.56	(0.26, 1.22)		1.04	(0.52, 2.10)		0.77	(0.40, 1.48)	
KES	15 kg (M), 11 kg (F)	2.22	(1.35, 3.66)	**	1.14	(0.58, 2.22)		1.49	(0.86, 2.56)		2.12	(1.17, 3.84)	*
POMA	24/25	0.95	(0.45, 2.01)		1.43	(0.66, 3.09)		1.06	(0.46, 2.41)		1.20	(0.58, 2.45)	
MMSE	≤23 vs. ≥24	1.13	(0.46, 2.77)		0.46	(0.23, 4.51)		0.97	(0.40, 2.37)		0.92	(0.47, 1.79)	
GDS	≥5 vs. <5	0.73	(0.22, 2.43)		0.84	(0.19, 3.62)		0.67	(0.16, 2.83)		1.15	(0.34, 3.89)	
Frailty Index	≥0.15 vs. <0.15	1.85	(1.03, 3.33)	*	2.13	(1.01, 4.51)	*	2.42	(1.30, 4.50)	**	1.00	(0.52, 1.93)	
Physical frailty	3–5 vs. 0–2	0.41	(0.13, 1.24)		1.18	(0.47, 2.94)		0.85	(0.28, 2.63)		0.62	(0.26, 1.47)	

Abbreviation: HR: hazard ratio; $^{*}p < 0.05$; $^{**}p < 0.01$; $^{***}p < 0.001$. All physical and functional performance measure was included in the same model together. Binary cut-offs shown are commonly used in previous research and clinical applications.

Supplementary Table 2. Sex- and age-stratified analyses of area under curve for physical and functional performance measures predicting mortality.

Measure	Men				Women				Age < 75				Age ≥ 75			
	**AUC**	**95%**	**CI**	***P***	**AUC**	**95%**	**CI**	***P***	**AUC**	**95%**	**CI**	***P***	**AUC**	**95%**	**CI**	***P***
TUG	0.737	0.684	0.790	***	0.741	0.662	0.820	***	0.712	0.649	0.775	***	0.619	0.542	0.696	**
GV (Reversed)	0.684	0.627	0.741	***	0.678	0.593	0.763	***	0.645	0.580	0.710	***	0.554	0.468	0.640	
KES	0.648	0.581	0.715	***	0.634	0.557	0.711	**	0.520	0.448	0.591		0.544	0.466	0.621	
POMA	0.550	0.495	0.605		0.577	0.501	0.652	*	0.531	0.476	0.586		0.540	0.468	0.612	
MMSE (Reversed)	0.618	0.553	0.683	***	0.699	0.617	0.781	***	0.575	0.508	0.643	*	0.572	0.488	0.655	
GDS	0.550	0.488	0.613		0.625	0.540	0.710	**	0.559	0.489	0.629		0.553	0.476	0.629	
Frailty Index	0.611	0.536	0.685	**	0.664	0.566	0.762	**	0.576	0.491	0.661		0.548	0.456	0.640	
Physical frailty	0.579	0.514	0.644	*	0.677	0.594	0.759	***	0.565	0.494	0.635		0.580	0.506	0.654	*
Age	0.696	0.634	0.759	**	0.750	0.669	0.832	***	0.650	0.579	0.721	**	0.555	0.470	0.640	

Abbreviation: AUC: area under curve; $^{*}p < 0.05$; $^{**}p < 0.01$; $^{***}p < 0.001$.

Supplementary Table 3. List of 98 binary variables used to derive frailty index.

1	History of hypertension
2	History of high cholesterol
3	History of diabetes
4	History of stroke
5	History of heart attack
6	History of atrial fibrillation
7	History of heart failure
8	History of eye problem
9	History of hearing loss
10	History of kidney failure
11	History of asthma
12	History of COPD
13	History of tuberculosis
14	History of arthritis
15	History of osteoporosis
16	History of hip fracture
17	History of neurodegenerative disorders
18	History of gastrointestinal problem
19	History of thyroid problem
20	History of cancer
21	History of depression
22	History of other mental disorders
23	History of dementia
24	History of Parkinson's disease
25	History of other neurological disorder
26	History of cancer
27	Illness/condition that changes the kind/amount of food eaten
28	Difficulty eating due to tooth/mouth problems
29	Unintended loss 4.5 kg in last 6 months
30	Difficulty in falling asleep
31	Frequent awakenings and difficulty in going back to sleep
32	Wake up very early and difficulty in going back to sleep
33	Feel tired in daytimes
34	Feel excessively sleepy in daytimes
35	Pain interfere with sleep
36	Poor sleep quality
37	Informant report of memory decline/mental ability
38	Less able to manage to find personal belonging at home
39	Less able to manage own finance
40	Less able to manage to keep appointment
41	Less able to read for over 5 minutes at a time
42	Cognitive impairment (age-education- adjusted MMSE < 23)
43	Poorer memory compared to other of similar age
44	Poorer memory/mental abilities compared to earlier period
45	Less able to remember things about family and friends (compared to 1 year ago)
46	Less able to remember things happened recently (compare to 1 year ago)
47	Less able to remember appointment and social arrangement (compared to 1 year ago
48	Less able to remember the place where things were put (compared to 1 year ago)
49	Less able to find way in getting around neighbourhood (compared with 1 year ago)
50	Less able to learn new things (compared to 1 year ago)
51	Less able to make decision on every matter (compared to 1 year ago)
52	Less able to follow news and understand what is going on (compared to 1 year ago)
53	Less able to handling money (compared to 1 year ago)
54	Less able to choose or use the right words (compared to 1 year ago)
55	Less able to concentrate (compared to 1 year ago)

56	Health is fair or poor
57	Health limited in doing moderate activities during typical day
58	Health limited in climbing stairs during typical day
59	Health limited in accomplishing lesser
60	Health limited in work/other activities
61	Accomplish less because of emotional problems
62	Can't do work or other activities well because of emotional problems
63	Pain interfere with normal work
64	Not felt calm and peaceful
65	Have lots of energy
66	Felt downhearted and low
67	Physical health/emotional problems interfere with social activities past 4 weeks
68	Have problem with Mobility
69	Have problem with Self-care
70	Have problem with Usual activity
71	Have Pain or discomfort moderate to extreme
72	Have moderate to extreme anxiety or depression
73	Life fairly to very boring
74	Life fairly to very sad
75	Life fairly to very hard
76	Very lonely
77	Depressive symptoms (GDS > = 5)
78	Hospitalization in past 1 year
79	Polypharmacy (> = 5 medication)
80	Bowel incontinence
81	Bladder incontinence
82	Dependent on personal grooming
83	Dependent on toilet use
84	Dependent on feeding
85	Dependent on transfer (from bed to chair & back)
86	Dependent on mobility (about the house)
87	Dependent on Dressing
88	Dependent on stairs climbing
89	Dependent on bathing
90	FEV1/FVC < 0.70
91	Chronic cough
92	Chronic sputum
93	Breathlessness
94	Body mass loss (BMI < 18.5 kg/m^2)
95	Slow gait
96	Weakness
97	Exhaustion
98	Low physical activity
